# An immune-adrenergic pathway induces lethal levels of platelet-activating factor in mice

**DOI:** 10.1038/s42003-024-06498-7

**Published:** 2024-06-29

**Authors:** Shuto Tanaka, Masataka Kawakita, Hikaru Yasui, Koichi Sudo, Fumie Itoh, Masato Sasaki, Nobuyuki Shibata, Hiromitsu Hara, Yoichiro Iwakura, Tomomi Hashidate-Yoshida, Hideo Shindou, Takao Shimizu, Taiki Oyama, Himawari Matsunaga, Kazuhiko Takahara

**Affiliations:** 1 Department of Animal Development and Physiology, Graduate School of Biostudies, Kyoto University, Kyoto, Japan; 2 Division of Infection and Host Defense, Tohoku Medical and Pharmaceutical University, Sendai, Japan; 3 Department of Immunology, Graduate School of Medical and Dental Sciences, Kagoshima University, Kagoshima, Japan; 4 Research Institute for Biomedical Sciences, Tokyo University of Science, Chiba, Japan; 5 Department of Lipid Life Science, National Center for Global Health and Medicine, Tokyo, Japan; 6 Department of Medical Lipid Science, Graduate School of Medicine, University of Tokyo, Tokyo, Japan; 7 Department of Lipid Signaling, National Center for Global Health and Medicine, Tokyo, Japan; 8 Institute of Microbial Chemistry, Tokyo, Japan

**Keywords:** Neuroimmunology, Neuroimmunology

## Abstract

Acute immune responses with excess production of cytokines, lipid/chemical mediators, or coagulation factors, often result in lethal damage. In addition, the innate immune system utilizes multiple types of receptors that recognize neurotransmitters as well as pathogen-associated molecular patterns, making immune responses complex and clinically unpredictable. We here report an innate immune and adrenergic link inducing lethal levels of platelet-activating factor. Injecting mice with toll-like receptor (TLR) 4 ligand lipopolysaccharide (LPS), cell wall N-glycans of *Candida albicans*, and the α_2_-adrenergic receptor (α_2_-AR) agonist medetomidine induces lethal damage. Knocking out the C-type lectin Dectin-2 prevents the lethal damage. In spleen, large amounts of platelet-activating factor (PAF) are detected, and knocking out lysophospholipid acyltransferase 9 (LPLAT9/LPCAT2), which encodes an enzyme that converts inactive lyso-PAF to active PAF, protects mice from the lethal damage. These results reveal a linkage/crosstalk between the nervous and the immune system, possibly inducing lethal levels of PAF.

## Abstract (web summary)

The authors show that stimuli with innate immune and adrenergic receptors induce lethal levels of platelet-activating factor in mice, suggesting a cross-talk between the immune and neural systems.

## Introduction

Acute, excess immune responses with an accompanying cytokine storm, vasopermeability, and thrombosis/coagulation frequently lead to multiple organ failure and lethal damage of the body^1^. Anaphylactic responses are categorized into two types, acquired and non-acquired immune response. The acquired type is caused by secondary responses such as IgE antibodies specific to antigens in food, insect venom, or various types of haptens^2^. Once specific antigens have been identified, it is often practicable for hypersensitive individuals to avoid them, or treat the responses with adrenaline. The non-acquired type is caused by innate immune responses, *e.g*., through C3a and C5a generated by the complement system, as well as direct activation of mast cells that release chemical mediators^3^. In addition, there may be additional, as yet unknown pathways in the non-acquired immune responses^3,4^, causing unanticipated anaphylactic responses to anesthetics/sedatives in medical operations.

Linkage/crosstalk between the nervous system and the immune system is crucial for homeostasis of the body in both the steady and the infected state^5^. For example, immune cells express several neuroreceptors^6^ and pattern recognition receptors (PRRs)^7^. The PRRs recognize various types of ligands originating from pathogens; ligands such as polysaccharides, peptidoglycans, lipoproteins, and nucleic acids elicit the propagation of diverse cellular signals^7^. Meanwhile, neurons also express some cytokine receptors^8^ and PRRs^9^. Given the aforementioned crosstalk between the nervous system and the immune system, simultaneous stimulation with neurotransmitters and PRR ligands generates complex immune responses, such as the alternation of cytokine and chemical mediator production from immune cells^6^.

In medical practice, the nervous system is routinely manipulated by anesthesia and sedation using drugs targeting neuroreceptors^10^, possibly altering immune responses through the immune-adrenergic crosstalk^11^. Anesthesia is considered to be a safe and established procedure for surgery^12^. However, rare incidents, including death, occur during anesthesia, with complications involving the circulatory system^13^, including pulmonary embolism and deep venous thrombosis^14^. Although the underlying mechanisms are still elusive, anesthetics targeting neuroreceptors on immune cells quite likely cause such side effects with thrombus formation. It is possible that the immune-adrenergic crosstalk might be especially consequential during anesthesia for infected/septic patients, because the patients often require general anesthesia for medical procedures, resulting in innate immune and adrenergic activation.

In the 1970’s, an immunosuppressive factor was found in the blood of candidiasis patients^15^. We previously showed that the immunosuppressive factors were N-glycans derived from surface mannoproteins of opportunistic candida strains^16^. These N-glycans consist of large numbers of mannoses with two N-acetylglucosamines at the reducing end in the mannose core. The linkages of mannoses are specific to strains, especially in their side chains, forming polysaccharide structures that are uniform for each strain^17^. We found that N-glycans from certain strains suppress the cytokine storm by the induction of immune suppressive cytokine IL-10 in the acute phase, and they protect antigen specific T cells from exhaustion in the late phase of sepsis^16^. In related experiments, we serendipitously found a high susceptibility of sepsis-induced mice with LPS and the N-glycan to the anesthetic medetomidine^18^ and its D-form enantiomer, dexmedetomidine, which are highly selective full agonists for α_2_-adrenergic receptor (α_2_-AR). Thus, we predicted an unknown crosstalk between innate immune and adrenergic system.

Here, we propose that crosstalk between the adrenergic and innate immune system results in the production of large amounts of platelet-activating factor (PAF) and subsequent lethal damage in mice. Our results show a plasticity of C-type lectin responses in the presence of α_2_-AR agonist, arguing for heightened clinical precautions in anesthesia and medication with α_2_-AR targeted medicines for septic and infected patient.

## Results

### Lethal damage with innate immune stimuli and anesthetics

**Fig. 1:**
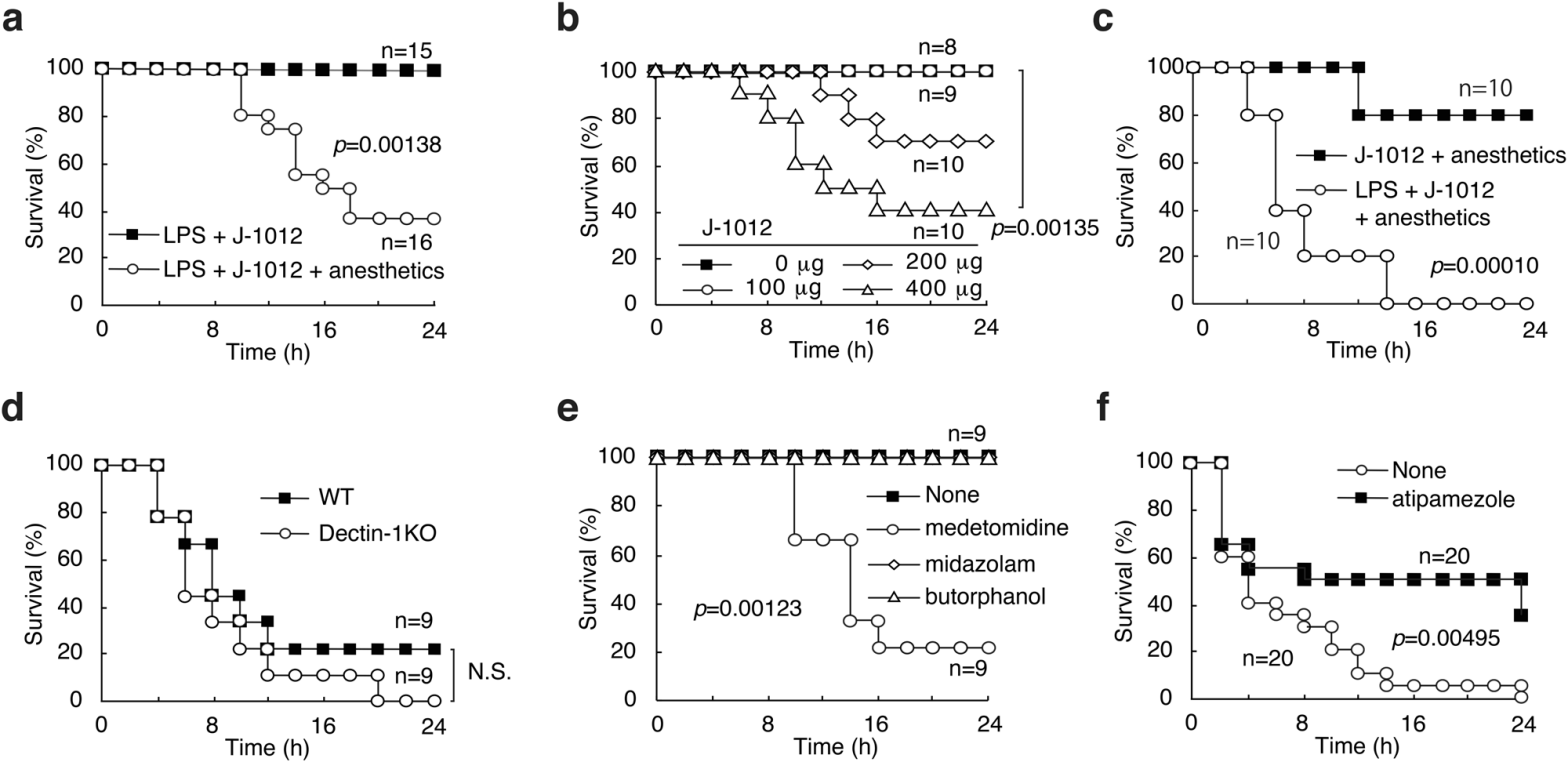
*C. albicans* N-glycan, LPS, and anesthetics induce mouse death. **a**–**f** Survival rates of Balb/c mice *i.v*. injected (**a**) with or without (*n* = 16 and *n* = 15 female mice, respectively) the mixed anesthetics (medetomidine 6 μg/20 g mouse body weight, midazolam 80 μg/20 g and butorphanol 100 μg/20 g) in the presence of LPS (15 µg/20 g) and J-1012 N-glycan (200 µg/20 g) (**b**) with phosphate buffered saline (PBS), J-1012 N-glycan (0, 100, 200 and 400 µg/20 g) in the presence of the mixed anesthetics and LPS (*n* = 8, 9, 10 and 10 female mice, respectively), (**c**) with or without LPS in the presence of J-1012 N-glycan (400 µg/20 g) and the mixed anesthetics (*n* = 10 female mice). **d** Survival rates of Balb/c and Dectin-1KO mice *i.v*. injected with the mixed anesthetics, LPS and J-1012 N-glycan (400 µg/20 g, *n* = 9 female mice). **e** Survival rates of Balb/c mice *i.v*. injected with PBS, medetomidine (18 µg/20 g), midazolam (240 µg/20 g) or butorphanol (300 µg/20 g) in the presence of LPS and J-1012 N-glycan (200 µg/20 g, *n* = 9 female mice), (**f**) with the mixed anesthetics, LPS and J-1012 N-glycan (400 µg/20 g) in the presence of atipamezole (12 μg/20 g) (*n* = 20 female mice). **a**–**f** All data shown are compiled from two or three independent experiments. Data were analyzed by the (**a**–**e**) Wilcoxon test and (**f**) Log Rank test. N.S. not significant.

Balb/c mice were susceptible to simultaneous injection of a mixture of anesthetics (medetomidine 6 μg/20 g mouse body weight, midazolam 80 μg/20 g and butorphanol 100 μg/20 g), ultrapure LPS (15 μg/20 g), and N-glycan (200 μg/20 g) purified from a surface mannoprotein of clinically isolated *C. albicans* strain J-1012 (hereinafter referred to as J-1012 N-glycan) (Fig. 1a). Death within 24 h of treatment was dose-dependent (Fig. 1b). Omitting LPS from the mixture increased survival significantly (Fig. 1c). Composition analysis of a J-1012 N-glycan preparation by gas chromatography detected contamination by small amounts (~3%) of glucose, possibly derived from β-glucan in the cell wall. However, mice lacking Dectin-1, which recognizes β-glucan^19^, were still susceptible (Fig. 1d). In wild type mice, separate injections of each anesthetic implicated medetomidine, a full agonist of α_2_-AR, in the susceptibility (Fig. 1e). Atipamezole, an antagonist of α_2_-AR, improved the survival rate in 24 h (Fig. 1f). Medetomidine seemed to suppress cytokine production in the early phase (2 h), but to prolong them in the late phase (6 and 8 h), compared to other anesthetics (Supplementary Fig. 1a). Atipamezole canceled the prolonged production of cytokines TNF-α, IL-6, IFN-γ and MCP-1, in the late phase (Supplementary Fig. 1b). These results suggest that medetomidine increased the susceptibility of mice to *C. albicans* J-1012 N-glycan, possibly through overproduction of cytokines.

### Involvement of Dectin-2 in the lethal damage

**Fig. 2:**
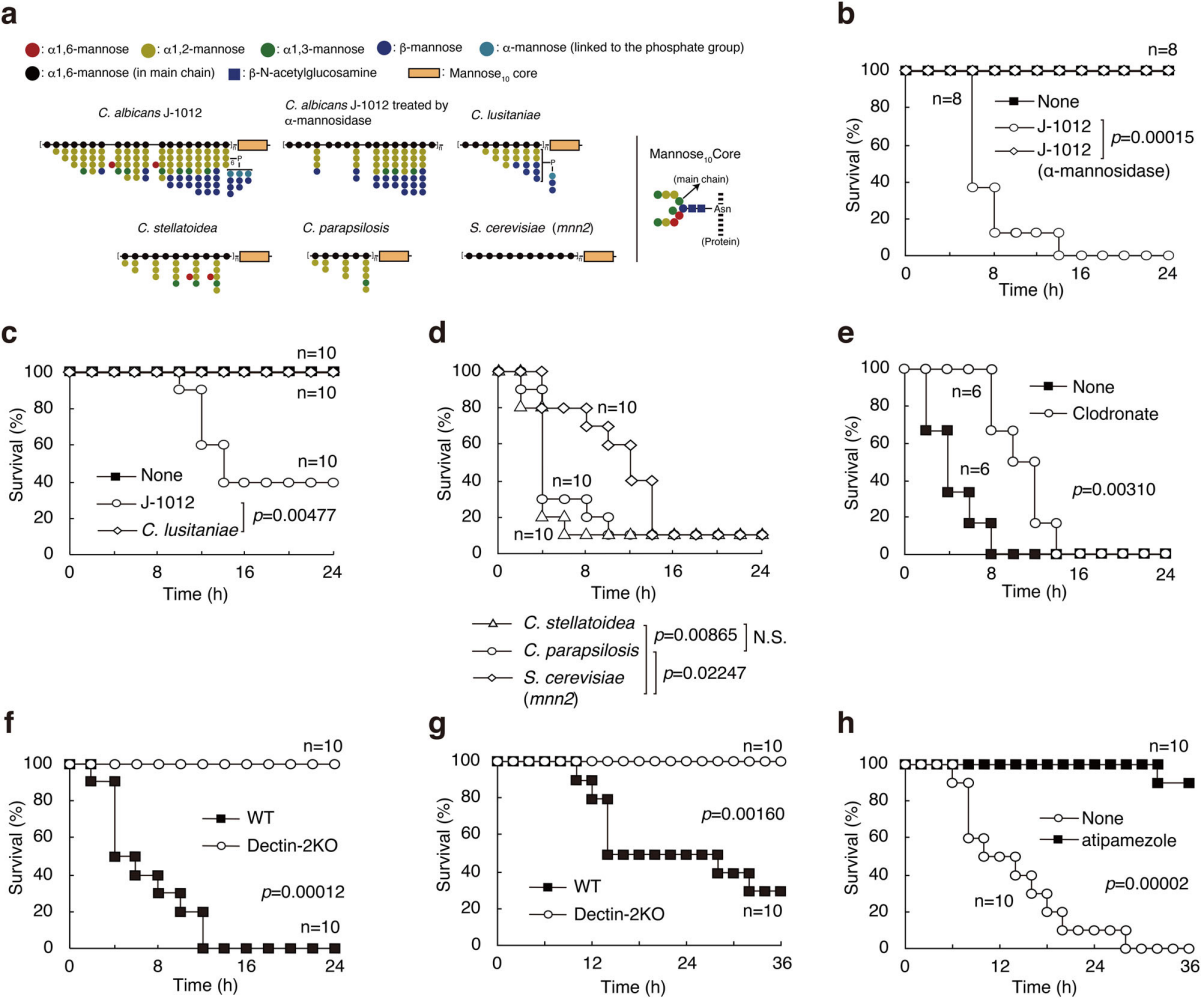
Dectin-2 is involved in the lethal damage. **a** Schematic structures of N-glycans and the mannose core: black circle, α1,6-mannose (in main chain); blue circle, β-mannose; green circle, α1,3-mannose; light blue circle, α-mannose (linked to the phosphate group); red circle, α1,6-mannose; yellow circle, α1,2-mannose; light brown rectangle, Mannose_10_ core). **b**–**d** Survival rates of Balb/c mice, treated with the mixed anesthetics and LPS, were *i.v*. injected with 400 μg/20 g of (**b**) J-1012 and α-mannosidase-treated J-1012 (*n* = 8 female mice), (**c**) *C. lusitaniae* (*n* = 10 female mice), (**d**) *C. stellatoidea*, *C. parapsilosis* and *S. cerevisiae* N-glycan (*n* = 10 female mice) with LPS (15 μg/20 g) and the mixed anesthetics. **e** Survival rates of Balb/c mice pre-treated with clodronate liposome were analyzed with LPS, the mixed anesthetics and 400 μg/20 g J-1012 N-glycan (*n* = 6 female mice). **f**, **g** Survival rates of Balb/c and Dectin-2KO mice were analyzed with (**f**) the mixed anesthetics (*n* = 7 male + 3 female mice) and (**g**) medetomidine (18 μg/20 g) with LPS and 400 μg/20 g J-1012 N-glycan (*n* = 10 female mice). **h** Survival rates of Balb/c mice *i.v*. injected with dexmedetomidine (9 μg/20 g), LPS, and *C. stellatoidea* N-glycan (200 μg/20 g) in the presence of atipamezole (36 μg/20 g) (*n* = 10 female mice). All data shown are compiled from two or three independent experiments. Data were analyzed by the Wilcoxon test.

We previously reported that J-1012 N-glycan (see Fig. 2a) contains various types of side chains: α-mannan side chains (55.4% of side chain in the number), β-mannan-capped α-mannan side chains (41.9%), and phosphomannan side chains (2.7%)^20^. It is possible that certain structural patterns in the side chains are involved in the mortality. We then checked various types of N-glycans that are structurally defined (Fig. 2a). Treatment of J-1012 N-glycan with α-mannosidase, which removes α-mannan side chains, leaving the mannose core/Man_10_GlcNAc_2_ and phosphomannan containing β-mannan side chain in J-1012 N-glycan, significantly decreased the mortality, suggesting that the α-mannosidase-sensitive polysaccharide patterns contribute to the J-1012 N-glycan’s lethality (Fig. 2b). N-glycan from *C. lusitaniae* (non-albicans strain), in which the side chains are predominantly β-mannan-capped α-mannan (77.5%) rather than α-mannan (14.9%) and phosphomannan (7.6%) chains^21^, did not cause any mouse deaths (Fig. 2c). By contrast, *C. stellatoidea* (*Candida albicans* (Robin) Berkhout, a synonym of *C. albicans* NBRC 1397) N-glycan without phosphomannan and β-mannose-capped side chains^22^ induced lethal damage (Fig. 2d). Moreover, non-albicans strain *C. parapsilosis*^23^, as well as *S. cerevisiae* (*mnn2*) lacking N-glycan side chains^24^, also provoked lethal damage (Fig. 2d). The *S. cerevisiae* (*mnn2*) strain still has the mannose_10_ core and the α1,6-mannose main chain, and it proved to be only slightly less lethal (merely delaying death by about 8-10 h) than *C. stellatoidea* and *C. parapsilosis*. These results strongly implicate the α-mannan structure of the *Candida*-like N-glycans in the lethal damage, irrespective of the particular *albicans* or non-*albicans* strain.

The pattern of N-glycan inducing the lethal damage seemed to be similar to the recognition pattern of C-type lectin Dectin-2^25^, a pattern recognition receptor for *C. albicans*^16,26^, on phagocytic cells. To explore the possible contribution of Dectin-2 to the lethal response of mice to N-glycan, we first depleted phagocytic cells in vivo by clodronate liposome and observed a decrease in susceptibility to N-glycan (Fig. 2e). We then tested Dectin-2 knockout (KO) mice, observing complete resistance to the damage provoked by the combination of LPS, N-glycan, and the anesthetic mixture (Fig. 2f). This was also the case with medetomidine (Fig. 2g). We next tested dexmedetomidine, the active enantiomer in the racemic mixture of medetomidine that is clinically used, with *C. stellatoidea* N-glycan containing only α-mannan side chains. The susceptibility of mice treated with *C. stellatoidea* N-glycan (200 μg/20 g), LPS (15 μg/20 g) and dexmedetomidine (9 μg/20 g) was suppressed by atipamezole (Fig. 2h). Collectively, our results suggest that Dectin-2 is involved in the lethal damage through recognition of α-mannans in the N-glycans.

### PAF production through Dectin-2

**Fig. 3:**
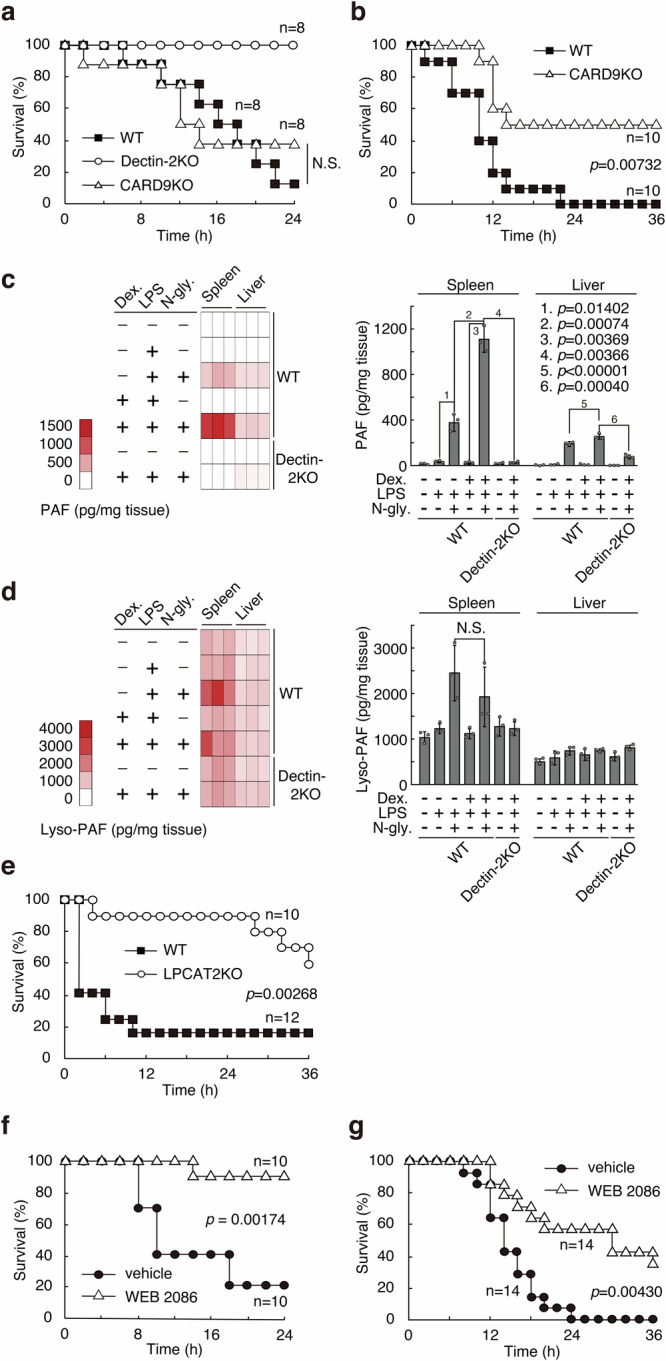
N-glycan of *C. albicans* upregulates production of platelet activating factor leading to the lethal damage. **a**, **b** Survival rates of Balb/c and CARD9KO and Dectin-2KO mice were analyzed with (**a**) the mixed anesthetics, LPS, and J-1012 N-glycan (400 μg/20 g, *n* = 4 male + 4 female mice), or (**b**) dexmedetomidine (9 μg/20 g) and *C. stellatoidea* N-glycan (200 μg/20 g, *n* = 5 male + 5 female mice). **c**, **d** Analyses of (**c**) PAF and (**d**) lyso-PAF in spleen and liver. Ten min after of injection with dexmedetomidine, LPS and *C. stellatoidea* N-glycan (200 μg/20 g), spleen and liver were analyzed by LC/MS (*n* = 3 female mice). **e** Survival rates of C57BL/6 (*n* = 4 male +8 female mice) and LPLAT9/LPCAT2KO (n = 3 male +7 female mice) mice after *i.p*. injection with dexmedetomidine and *i.v*. injection with LPS and *C. stellatoidea* N-glycan (200 μg/20 g). **f**, **g** Balb/c mice were injected with WEB 2086, and survival rates of the mice were analyzed with (**f**) J-1012 N-glycan (400 μg/20 g) and the mixed anesthetics (*n* = 10 female mice) or (**g**) *C. stellatoidea* (200 μg/20 g) N-glycan and dexmedetomidine (*n* = 14 female mice). **a**, **b**, **e**, **f**, **g** All data shown are compiled from two or three independent experiments by the Wilcoxon test. N.S., not significant. **c**, **d** Experiments were repeated twice, and representative results are shown. Data were analyzed by Student’s *t*-test, and expressed as the mean ± standard deviation (SD). N.S. not significant.

Upon ligand recognition, a signal from Dectin-2 is transduced *via* Syk kinase through CARD9, a member of the signalosome, together with BCL10 and Malt1. The signaling ultimately leads to activation of NF-κB and subsequent cytokine production^27,28^. Dectin-2 signaling also activates the arachidonate cascade *via* Syk^29^, possibly leading to a feed-forward loop to produce some cytokines. However, this might be a marginal contribution, because deletion of CARD9 resulted in significant decreases in IL-2, IL-10 and TNF-α upon stimulation of Dectin-2^30^. It is also reported that G protein-coupled α_2_-AR releases the Gαi subunit upon agonist binding^31^, resulting in NF-κB activation through phosphatidylinositol-3 kinase (PI_3_K)^32^. Thus, these pathways may co-operatively enhance cytokine production/cytokine storm^29^. Surprisingly, however, CARD9KO mice were still susceptible to the lethal damage provoked by the combination of LPS, N-glycan and the anesthetic mixture (Fig. 3a). In contrast, knocking out CARD9 suppressed to some extent the lethal damage provoked by the combination of LPS, *C. stellatoidea* N-glycan and dexmedetomidine (Fig. 3b), suggesting that an unknown effector may play a role in the susceptibility, apart from cytokines directly induced by Dectin-2 signaling.

Regarding Dectin-2 signaling, as mentioned above, ligand binding induces arachidonate, *e.g*., cysteinyl-leukotriene (Cys-LT) production^33^. In this pathway, it is supposed that cytosolic phospholipase A2 (cPLA2) hydrolyzes phosphatidylcholine into arachidonate and lysophosphatidylcholine, leading to Cys-LT and PAF, respectively. PAF is a potent phospholipid activator and mediator that induces anaphylactic responses^34^, neuropathic pain^35^, and several inflammatory events^36,37^. We therefore looked for enhanced production of PAF in our experimental conditions. We first quantified PAF in vivo using liquid chromatography/mass spectrometry (LC/MS)^38,39^, observing its upregulation in spleen and liver upon injection with LPS and the N-glycan, in comparison with LPS (Fig. 3c, Supplementary Fig. 2).

PAF receptor (PAF-R) is a G protein-coupled receptor (GPCR)^37,40^. There are major and minor types of PAF-R coupled with Gq and Gαi subunits, respectively. The Gq subunit with the major PAF-R directly causes a positive feedback loop (PAF loop), whereas the Gαi subunit with the minor PAF-R indirectly upregulates PAF production by blocking the cyclic AMP (cAMP)-PKA pathway suppressing the PAF loop^41^. Like the minor PAF-R, α_2_-AR is also a GPCR coupled with the Gαi subunit^31^. Therefore, we expected that the α_2_-AR Gαi subunit would counter the PKA-dependent PAF loop regulatory pathway. We checked production of PAF and its precursor lyso-PAF in response to LPS and the N-glycan in the presence of dexmedetomidine. Interestingly, PAF in spleen significantly increased with dexmedetomidine (Fig. 3c). A combination of LPS and dexmedetomidine didn’t upregulate PAF production in comparison with LPS alone, suggesting that the PAF induced by LPS stimulation is insufficient to initiate the PAF loop. In addition, PAF, but not lyso-PAF production was suppressed in vivo in Dectin-2KO mice, suggesting that PAF induced by the N-glycan and LPS is sufficient to start the PAF loop with dexmedetomidine. Production of lyso-PAF was upregulated in spleen by LPS and the N-glycan (Fig. 3d). However, this elevated lyso-PAF production wasn’t upregulated by the further addition of dexmedetomidine, possibly because of robust change from lyso-PAF to PAF. Therefore, it is possible that PAF is initially generated through the N-glycan-Dectin-2 axis, and is amplified to the lethal levels by the dexmedetomidine-α_2_-AR axis. In liver, lyso-PAF levels did not increase with dexmedetomidine treatment in the presence of N-glycan and LPS.

To confirm an involvement of PAF in the lethal damage, we examined mice with a knock-out of the gene encoding lysophospholipid acyltransferase 9 (LPLAT9, also called LPCAT2)^35^, which converts latent lyso-PAF to active PAF in the final step of PAF synthesis, and observed reduced susceptibility to the damage (Fig. 3e). On the other hand, it was reported that LPLAT9/LPCAT2 acetylates TLR4 and translocates into lipid rafts, enhancing cytokine production in RAW264.7 cells^42^. However, the loss of LPLAT9/LPCAT2 only slightly affected serum cytokines upon injection of LPS in vivo (Supplementary Fig. 3).

We next tested PAF-R antagonist WEB 2086. Pre-injection of the antagonist significantly ameliorated the susceptibility of mice to *C. albicans* J-1012 N-glycan and the anesthetic mixture (Fig. 3f), or to *C. stellatoidea* N-glycan and dexmedetomidine (Fig. 3g) in the presence of LPS, again implicating PAF in the lethal damage. It was reported that PAF induces IL-1β in a NLRP3-NEK7 inflammasome-dependent, but a PAF-R-independent manner^43^. However, our results using WEB 2086 suggest that PAF is involved in the lethal damage through PAF-R, but not the NLRP3-NEK7 pathway. Taken together, our results suggest that PAF generated by Dectin-2 and LPS causes the non-acquired anaphylactic death in the presence of an α_2_-AR agonist.

### Effects of another TLR ligand

**Fig. 4:**
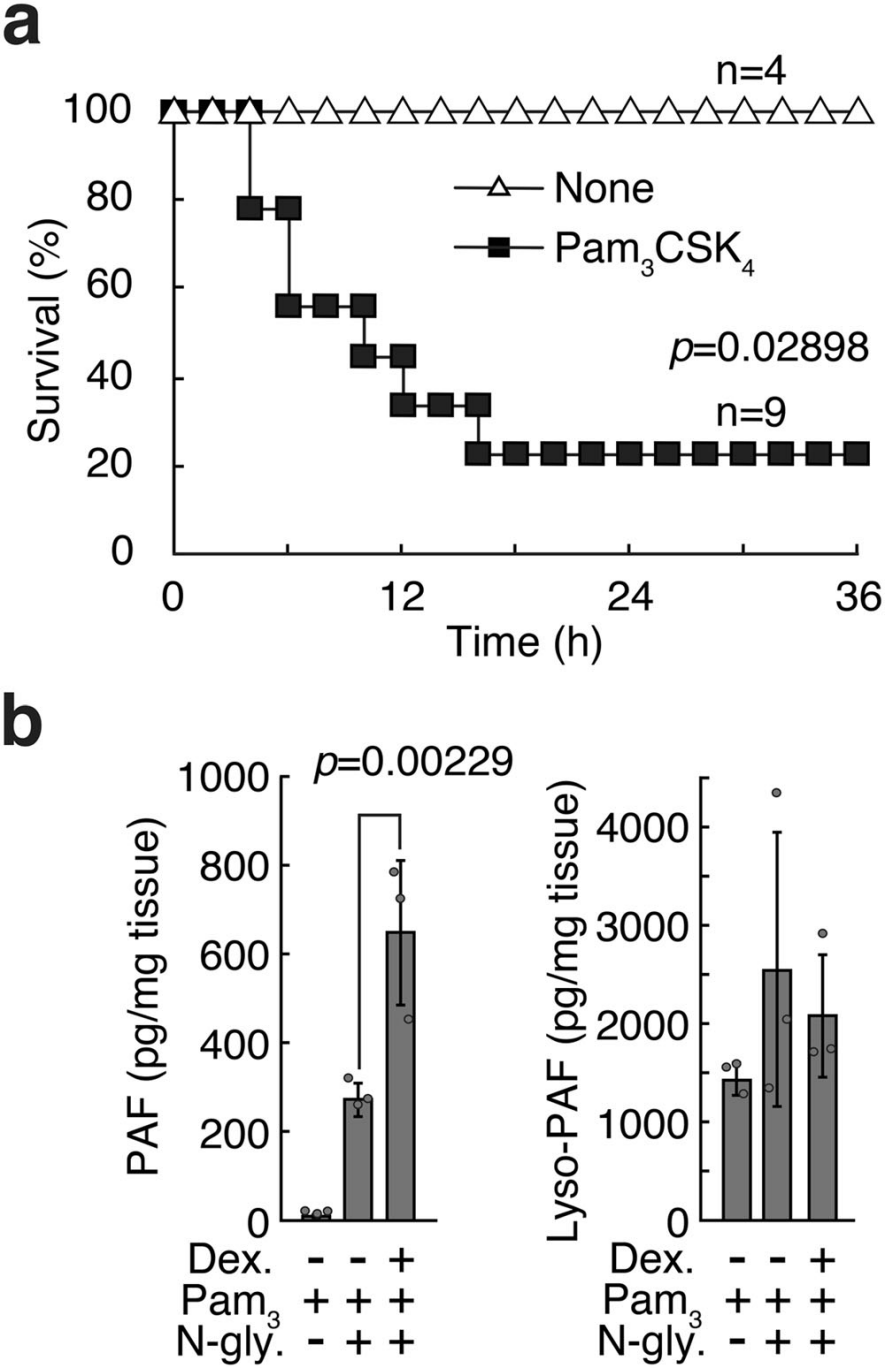
PAF induction by another TLR ligand. **a** Survival rates of Balb/c mice were analyzed with (*n* = 9 female mice) or without (*n* = 4 female mice) Pam_3_CSK_4_ (15 μg/20 g), in the presence of the mixed anesthetics and *C. stellatoidea* N-glycan (200 μg/20 g). Data shown are compiled from two independent experiments, and were analyzed by the Wilcoxon test. **b** Analyses of PAF and lyso-PAF in spleen treated with LPS dexmedetomidine, LPS and *C. stellatoidea* N-glycan (200 μg/20 g) as in Fig. 3c (*n* = 3 female mice). Experiments were repeated twice, and representative results are shown. Data were analyzed by Student’s *t*-test, and expressed as the mean ± SD.

We next tested another TLR ligand. Pam_3_CSK_4_, a synthetic TLR2 ligand, also induced lethal damage in combination with the N-glycan and the anesthetic mixture (Fig. 4a), with the upregulation of PAF production in the spleen (Fig. 4b). It is known that TLR2 signals are transduced by MyD88 and the MyD88+TRAM pathway^44^, leading to the induction of proinflammatory cytokines and type I interferon production, respectively. However, it seems that Pam_3_CSK_4_ doesn’t activate the MyD88+TRAM pathway^45^, suggesting that the MyD88 pathway is sufficient for PAF induction.

## Discussion

We propose that a link between the innate immune and adrenergic system results in the production of a large, deleterious amount of PAF (Supplementary Fig. 4). The innate stimuli through Dectin-2 and TLR2/4 generate initial PAF production, which ignites the PAF loop. Subsequently, the Gαi subunit released from α_2_-AR may drive the PAF loop further. We think that the ‘ignition-drive’ action leads to the lethal damage in mice.

The augmentation of PAF production requires both the innate immune and the α_2_-AR adrenergic stimuli, so that PAF levels are normally kept in check. However, during medical operations/treatments, a common situation might entail the activation of the innate immune system and α_2_-AR system by pathogen-associate molecular patterns (PAMPs)/damage-associated molecular patterns (DAMPs)/altered-self ligands^46^ and by administration of α_2_-AR agonists, respectively. Furthermore, certain anesthetics^47^ cause anaphylactic responses. At present, it is difficult to distinguish these responses from classical anaphylaxis caused by acquired immunity, which marshals antibodies specific to excipients in the medicines^48^, but it is likely that some of the anaphylactic responses upon use of anesthetics are caused by antigen non-specific responses without sensitization. Our finding supports a plausible mechanistic explanation for the non-acquired type of anaphylactic responses.

Dexmedetomidine is widely used clinically for several medical protocols as well as for anesthesia. α_2_-AR, on which dexmedetomidine acts, is a unique AR that negatively controls neural transmission, which makes it useful target of drugs for a broad range of conditions, *e.g*., hypertension (methyldopa hydrate, clonidine hydrochloride), glaucoma (brimonidine tartrate), spasm, multiple sclerosis/MS, muscular atrophic lateral sclerosis/ALS (tizanidine hydrochloride) and attention defect hyperactivity disorder/ADHD (guanfacine hydrochloride, clonidine hydrochloride) treatment. Based on our study, the same cautions as for dexmedetomidine may be required for these therapeutic agents.

Looking further into the ignition of the PAF loop, TLR ligands, LPS and Pam_3_CSK_4_ both induced PAF in vivo in the presence of the N-glycan and dexmedetomidine, suggesting that the Myd88 pathway may serve as a nexus in the PAF induction. Actually, LPLAT9/LPCAT2 is activated by PAF-R^49^ and TLR4 stimulations^50^. If so, TLR5, 7, 8, 9 using MyD88^51^ might be involved in the PAF production. On the other hand, using the sepsis model, we previously found immuno-modulating activities of the N-glycans^16^ that were reported in blood circulation of candidiasis patients infected with the opportunistic pathogen *C. albicans*^52^. In this case, the N-glycan induced IL-10 production and subsequent suppression of the cytokine storm in a Dectin-2-dependent manner in vivo^16^. In contrast, with the addition of medetomidine, the N-glycans augmented the production of proinflammatory cytokines and PAF, suggesting a degree of plasticity of innate responses by the N-glycan-Dectin-2 axis.

Medetomidine and other α_2_-AR targeting anesthetics are often used in experiments/treatments with animals. Such anesthetics possibly affect cytokine and PAF production. Therefore, we should consider such side-effects in the interpretation of animal experiments that make use of anesthetics.

PAF is produced in a balance with a remodeling pathway of glycerophospholipids (Lands’ cycle)^53^. At present, it isn’t clear whether the lethal levels of PAF are derived from efficient conversion of lyso-PAF to PAF or inefficient conversion of PAF to lyso-PAF. Furthermore, the amounts of lyso-PAF derived from alkyl-phosphatidylcholine are also increased by cPLA2 during PAF production, possibly affecting the level of PAF. Moreover, the activities of cPLA2 and LPLAT9/LPCAT2 are Ca^2+^-dependent^54^. However, Dectin-2 and TLR signaling do not seem to upregulate Ca^2+^ directly. Therefore, it is possible that Ca^2+^ mobilization caused by α_2_-AR signaling^55^ augments the levels of PAF in addition to the suppression of the cAMP-PKA axis. However, this Ca^2+^ mobilization by the Gαi-Gβγ subunit of α_2_-AR requires Gq co-activation, possibly derived from Gq-coupled PAF-R.

It has also been reported that exogenous PAF injected *i.p*. protects mice from LPS sepsis^56^. However, in this case, the injected PAF may quickly be converted to lyso-PAF, which inhibits PAF^57^, by PAF acetylhydrolase (PAF-AH), which is ubiquitous in the body. On the other hand, intact/endogenous PAF works locally in the vicinity of its producer cells, due to the surrounding PAF-AH activity. Therefore, it is conceivable that PAF shows diverse functions depending on the experimental conditions; *e.g*., the method of administration/induction and localization in vivo.

The duration/time point of cytokine/PAF induction is long before the actual time of death in some cases. For example, with LPS injection in mice, peaks of proinflammatory cytokines TNF-α, IL-1β and IFN-γ were 1 h, 2 h and 6 h, respectively, and all mice died between 1 and 3 days after injection^16^. In addition, transient induction (~2 h) of immunosuppressive cytokine IL-10 led to a reduction in the number of mouse deaths^16^, and in this study PAF was induced within several minutes. These results suggest that early production of cytokines/PAF affect organ failure and result in death at a later stage. Local action and a second cycle of inflammation with cytokine production of PAF might cause this delay more significantly. This delay also makes it difficult to exactly distinguish between death by anaphylaxis versus a cytokine storm. However, LPLAT9/LPCAT2KO mice succumb by lethal production of PAF. Therefore, we think that the death is caused by an anaphylactic-like response mediated by PAF.

In future experiments, we would like to know whether the crosstalk affects PAF production under stressed conditions that induce hormones/neurotransmitters such as adrenaline and noradrenaline^58,59^, and under infected conditions with innate ligands from pathogens. It is also unknown which cell types and organs are involved in PAF production. This information will give us clues to fit our findings to human anaphylactic responses.

## Materials and methods

### Ethics statement

All animal experiments were approved by the Animal Research Committee, Graduate School of Biostudies, Kyoto University (protocol number Lif-K20008). We have complied with all relevant ethical regulations for animal use.

### Mice

Balb/c and C57BL/6 mice were obtained from Japan SLC (Hamamatsu, Shizuoka, Japan). Dectin-2KO^26^, CARD9KO^60^ and LPLAT9 (LPCAT2) (loxP) (Acc.No.CDB0649K, https://large.riken.jp/distribution/mutant-list.html)^35^ mice have been described previously. Mice were maintained under specific pathogen-free conditions and were used at 8–12 weeks of age. Female mice were mainly used in this study. In some cases, we compiled data using male and female mice as described in figure legends.

### Glycans from yeast

Cell wall mannoprotein was extracted from the acetone-dried cells using deionized water at 120 °C for 2 h. The extract was dialyzed against water, concentrated, and Fehling’s solution^20^ was added while stirring. After 5 min, the insoluble copper chelate of the mannan was collected by centrifugation. The copper ion in the complex was then removed using a cation-exchange resin (Amberlite IR120, H^+^ form) (Sigma-Aldrich, St. Louis, MO). The resultant glycan (mannan) solution was dialyzed and lyophilized^22^. The glycans used in this study were purified from strains of *C. albicans* J-1012 (*Candida albican*s (Robin) Berkhout, NBRC1060)^20^, *C. stellatoidea* (*Candida albican*s (Robin) Berkhout, NBRC 1397)^22^ and *C. lusitaniae* (non-*albicans* strain, NBRC1019)^21^. The α-mannosidase treatment of J-1012 N-glycan was carried out in 50 mM sodium acetate buffer (pH 4.6) containing 20 units of α-mannosidase (EC3.2.1.24) (Sigma-Aldrich, St. Louis, MO) at 37 °C for 48 h.

### Anesthetics and sepsis induction

The anesthetic mixture, consisting of medetomidine (6 μg/20 g body weight, Domitor, Nippon Zenyaku Kogyo, Tokyo, Japan), midazolam (80 μg/20 g, Dormicum, Astellas Pharma, Tokyo, Japan) and butorphanol (100 μg/20 g, Butorphale, Meiji Seika, Tokyo, Japan) in distilled water, was used as described in ref. ^61^. Dexmedetomidine was obtained from Nipro (Tokyo, Japan). When each anesthetic was administered alone, a concentration three-times what was in the mixture was used. α_2_-AR antagonist (Antisedan, Nippon Zenyaku Kogyo, Tokyo, Japan) was used at two- and four-times the dose of medetomidine and dexmedetomidine, respectively, in the anesthetic solution. To induce anesthesia, mice were *i.p*. administered 200 μL of the mixture per 20 g mouse body weight. For endotoxin shock, mice were injected *i.v*. with HANKS’ balanced salt solution (HBSS) with Ca^2+^ and Mg^2+^ (200 µl/20 g mouse body weight) containing 15 µg/20 g of ultrapure LPS from *E. coli* LPS (0111:B4, Invivogen, San Diego, CA) with 100 ~ 400 μg/20 g of glycans after 20 min. In additional experiments, 15 µg/20 g of Pam_3_CSK_4_ (Calbiochem, San Diego, CA) was used instead of LPS.

### Analyses of cytokine production

Cytokines in serum/culture supernatants were assessed by a Cytometric Bead Array (CBA) mouse inflammatory kit (BD Biosciences, Franklin Lakes, NJ) in accordance with the manufacturer’s protocols using a FACSCalibur system (BD Biosciences).

### Treatment of mice with clodronate liposomes

To deplete phagocytic cells in vivo, mice were treated *i.v*. and *i.p*. with anionic clodronate liposomes-A (FormuMax Scientific, CA) (60 μl/20 g body weight, respectively; total 120 μl)^16^. After 2 days, anesthetic death was induced with the aforementioned protocol.

### Analyses of PAF production

Spleen and liver were obtained 10 min after of injection. Frozen tissues were homogenized using the Automill (Tokken. Inc., Chiba, Japan), and then lipids were extracted from powder tissues using methanol adding PAF-d4 and lyso-PAF-d4 (Cayman Chemical, Ann Arbor, MI) as an internal standard. The samples were entrapped in an Oasis HLB solid-phase extraction cartridge (Waters, Milford, MA) equilibrated with 60% methanol and 0.03% HCHO. After washing with the equilibration solution and petroleum ether, lipid fraction containing PAF and lyso-PAF was eluted with 0.2% HCHO in methanol. PAF analyses were performed by liquid chromatography-tandem mass spectrometry (LC-MS/MS) (LCMS-8060, Shimazu Corporation, Kyoto, Japan) as described previously in refs. ^38,39^. PAF-C16 was detected as an acetyl fragment (m/z59.1) derived from degradation of PAF-C16 ([M + HCOOH-H]^-^, m/z568.4), avoiding overlap with PC(18:0/0:0) = LPC18:0. SRM transitions were 568.4 → 59.1 for PAF-C16, 572.4 → 59.1 for PAF-C16-d4, 482.3 → 104.2 for Lyso-PAF, 486.3 → 104.2 for Lyso-PAF-d4.

### Inhibition of PAF-R

To inhibit PAF-R, a PAF-R antagonist WEB 2086 (20 µg/ 20 g mouse weight, Enzo Life Sciences, Farmingdale, NY) in HBSS was *i.v*. administered just before injection with the anesthetics.

### Statistics and reproducibility

Data are expressed as the means of assays. Statistical significance was determined by the two-tailed Student’s *t*-test. Differences in the survival of each group were determined by the Wilcoxon test or Log Rank test. Number of mice and experiments are shown in each figure and respective legend. For some experiments, compiled results are shown.

### Reporting summary

Further information on research design is available in the Nature Portfolio Reporting Summary linked to this article.

### Supplementary information

Supplementary Information

Description of Additional Supplementary Files

Supplementary Data

Reporting Summary

## Data Availability

All raw data are stored and securely backed-up and available upon request. All data underlying the graphs are available as Supplementary Data. Reagents are available on reasonable request from the corresponding author.
