# Supplementary material for: An immune-adrenergic pathway induces lethal levels of platelet-activating factor in mice
Source: Commun Biol. 2024 Jun 29;7:782. doi: 10.1038/s42003-024-06498-7 (PMC11217416; doi:10.1038/s42003-024-06498-7)
Supplement: Supplementary file 1 — Supplementary Information [file 42003_2024_6498_MOESM1_ESM.pdf]

Supplementary Information

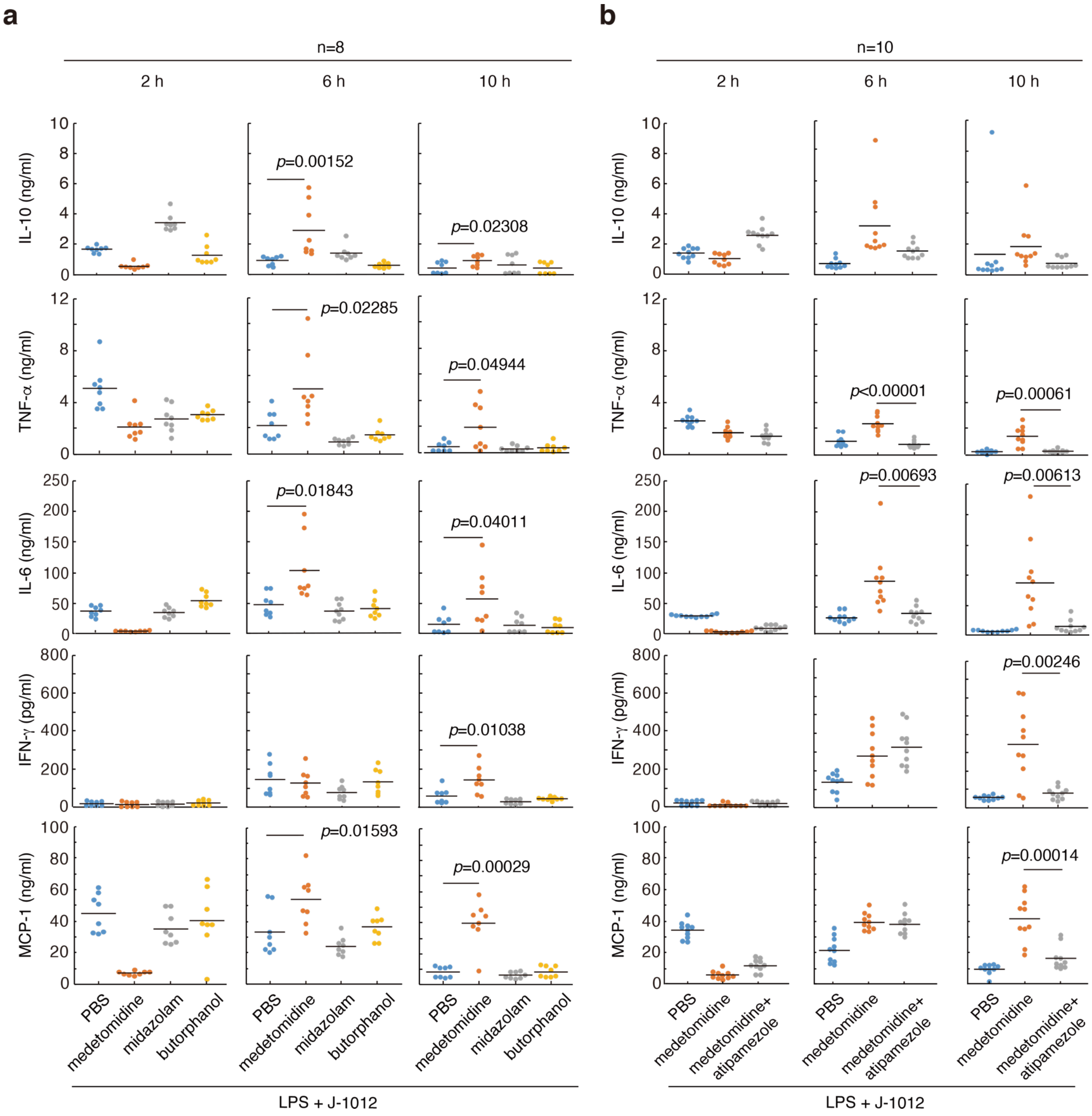

Supplementary Figure 1. Serum cytokine in mice treated with individual anesthetics and atipamezole.

Serum cytokine production in mice treated with **(a)** each anesthetic (medetomidine 18 μg, midazolam 240 μg, or butorphanol 300 μg per 20g body weight, respectively) (n=8 female mice), or with **(b)** medetomidine (18 μg/20 g) and atipamezole (36 μg/20 g) (n=10 female mice) in the presence of LPS and J-1012 N-glycan (400 μg/20 g) as in Fig. 1a. The data are

compiled from two independent experiments. Data were analyzed by Student's *t*-test, and expressed as the mean.

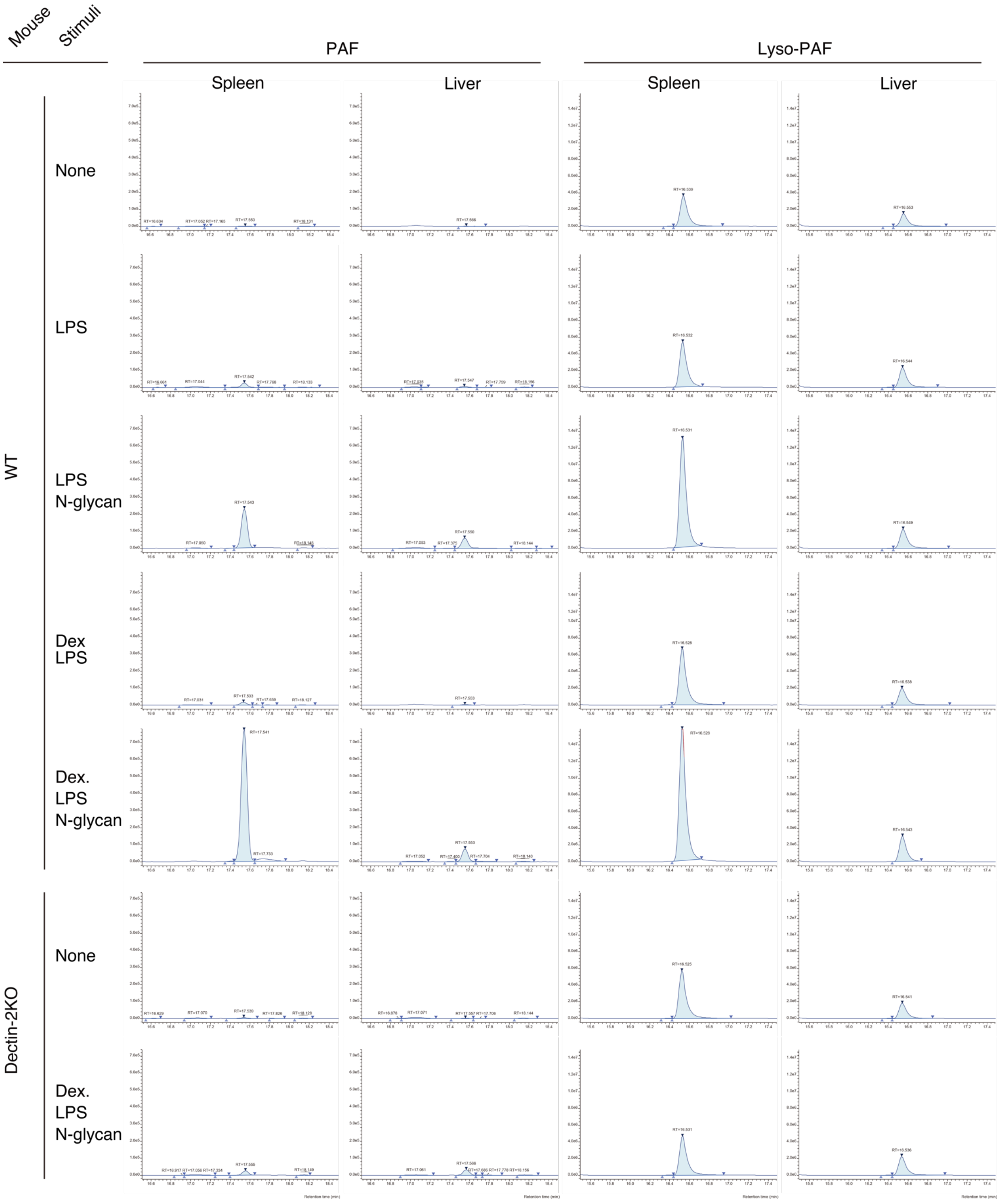

Supplementary Figure 2. Typical chromatograms of PAF and lyso-PAF in LC/MS analyses.

Ten min after injection with dexmedetomidine, C. stellatoidea N-glycan (200 μg/20 g) and LPS in combinations as indicated, lipids in the spleen and liver were extracted with methanol, and analyzed acetyl fragment (m/z59.0) derived from degradation of PAF-C16 ($[M+HCOOH-H]^-$, m/z568.3) by LC/MS with internal controls. RT, retention time.

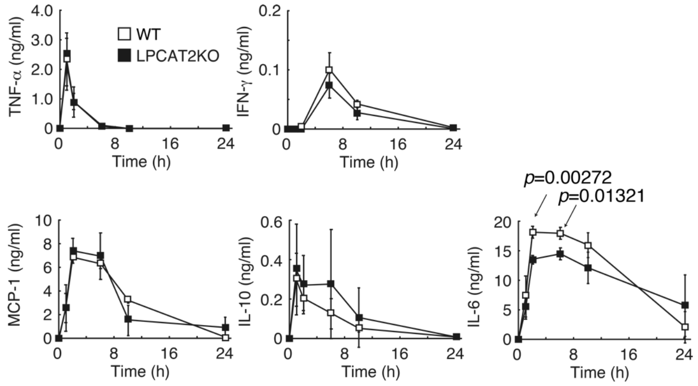

Supplementary Figure 3. Cytokine production in LPLAT9/LPCAT2KO mice upon injection of LPS.

Serum cytokines in C57BL/6 and LPLAT9/LPCAT2KO mice after injection of LPS (15 μg /20 g) (n=3 female mice). Experiments were repeated twice, and representative results are shown. Data were analyzed by Student's *t*-test, and expressed as the mean ± SD.

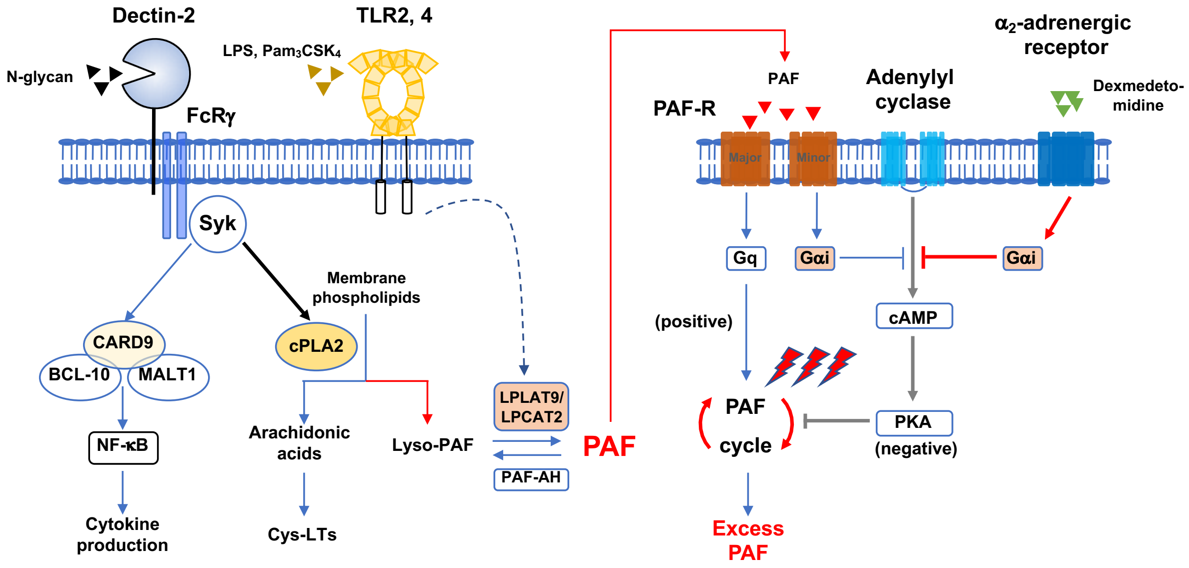

Supplementary Figure 4. Possible mechanism of PAF induction by Dectin-2 and α_2-AR crosstalk.

Signals form Dectin-2 are transduced *via* two pathways, Syk-CARD9 and Syk-cPLA2, leading to cytokine production and cysteinyl leukotrienes, respectively. In the latter pathway, it is thought that cPLA2 generates lyso-PAF. Subsequently, the lyso-PAF is converted to active PAF by LPLAT9/LPCAT2. The LPS-TLR4 pathway activates LPLAT9/LPCAT2 by its phosphorylation. The Pam_3CSK_4-TLR2 pathways are also involved in PAF production. On the other hand, there are two types of G protein coupled receptor, major and minor, for PAF signaling. Upon recognition of initial PAF, possibly generated *via* Dectin-2, the major PAF-R releases the Gq subunit, leading to further PAF production in what is called the PAF loop. Simultaneously, the other (minor) PAF-R releases the Gαi subunit, which suppresses adenylyl cyclase activity. This leads to downregulation of cAMP levels and subsequent PKA activity, thereby weakening negative regulation of the PAF loop by the cAMP-PKA pathway.

Therefore, initial PAF is amplified by the PAF loop with the Gq and Gαi subunits released from the respective PAF-Rs. α_2-AR is also a G protein coupled receptor with a Gαi subunit. Therefore, it is thought that dexmedetomidine also weakens the negative regulation by the cAMP-PKA pathway *via* α_2-AR, further augmenting the PAF loop ignited by Dectin-2-TLR signaling.
