# Supplementary material for: An immune-adrenergic pathway induces lethal levels of platelet-activating factor in mice
Source: Commun Biol. 2024 Jun 29;7:782. doi: 10.1038/s42003-024-06498-7 (PMC11217416; doi:10.1038/s42003-024-06498-7)
Supplement: Supplementary file 2 — Description of Additional Supplementary Files [file 42003_2024_6498_MOESM2_ESM.docx]

Description of Additional Supplementary Files

File name: Supplementary Data

Description: The source data behind the graphs in the paper
