# Supplementary material for: An immune-adrenergic pathway induces lethal levels of platelet-activating factor in mice
Source: Commun Biol. 2024 Jun 29;7:782. doi: 10.1038/s42003-024-06498-7 (PMC11217416; doi:10.1038/s42003-024-06498-7)
Supplement: Supplementary file 4 — Reporting Summary [file 42003_2024_6498_MOESM4_ESM.pdf]

nature portfolio

Corresponding author(s): Kazuhiko Takahara

Last updated by author(s): Jun 13, 2024

Reporting Summary

Nature Portfolio wishes to improve the reproducibility of the work that we publish. This form provides structure for consistency and transparency in reporting. For further information on Nature Portfolio policies, see our Editorial Policies and the Editorial Policy Checklist.

Statistics

For all statistical analyses, confirm that the following items are present in the figure legend, table legend, main text, or Methods section.

n/a	Confirmed	
☐	☒	The exact sample size (*n*) for each experimental group/condition, given as a discrete number and unit of measurement
☐	☒	A statement on whether measurements were taken from distinct samples or whether the same sample was measured repeatedly
☐	☒	The statistical test(s) used AND whether they are one- or two-sided<br>*Only common tests should be described solely by name; describe more complex techniques in the Methods section.*
☒	☐	A description of all covariates tested
☒	☐	A description of any assumptions or corrections, such as tests of normality and adjustment for multiple comparisons
☐	☒	A full description of the statistical parameters including central tendency (e.g. means) or other basic estimates (e.g. regression coefficient) AND variation (e.g. standard deviation) or associated estimates of uncertainty (e.g. confidence intervals)
☐	☒	For null hypothesis testing, the test statistic (e.g. *F*, *t*, *r*) with confidence intervals, effect sizes, degrees of freedom and *P* value noted<br>*Give P values as exact values whenever suitable.*
☒	☐	For Bayesian analysis, information on the choice of priors and Markov chain Monte Carlo settings
☒	☐	For hierarchical and complex designs, identification of the appropriate level for tests and full reporting of outcomes
☒	☐	Estimates of effect sizes (e.g. Cohen's *d*, Pearson's *r*), indicating how they were calculated

Our web collection on statistics for biologists contains articles on many of the points above.

Software and code

Policy information about availability of computer code

Data collection	Not applicable
Data analysis	Not applicable

For manuscripts utilizing custom algorithms or software that are central to the research but not yet described in published literature, software must be made available to editors and reviewers. We strongly encourage code deposition in a community repository (e.g. GitHub). See the Nature Portfolio guidelines for submitting code & software for further information.

Data

Policy information about availability of data

All manuscripts must include a data availability statement. This statement should provide the following information, where applicable:

- Accession codes, unique identifiers, or web links for publicly available datasets
- A description of any restrictions on data availability
- For clinical datasets or third party data, please ensure that the statement adheres to our policy

All data underlying the graphs are available as Supplementary Information.

Research involving human participants, their data, or biological material

Policy information about studies with human participants or human data. See also policy information about sex, gender (identity/presentation), and sexual orientation and race, ethnicity and racism.

Reporting on sex and gender	Not applicable
Reporting on race, ethnicity, or other socially relevant groupings	Not applicable
Population characteristics	Not applicable
Recruitment	Not applicable
Ethics oversight	Not applicable

Note that full information on the approval of the study protocol must also be provided in the manuscript.

Field-specific reporting

Please select the one below that is the best fit for your research. If you are not sure, read the appropriate sections before making your selection.

☒ Life sciences ☐ Behavioural & social sciences ☐ Ecological, evolutionary & environmental sciences

For a reference copy of the document with all sections, see nature.com/documents/nr-reporting-summary-flat.pdf

Life sciences study design

All studies must disclose on these points even when the disclosure is negative.

Sample size	The determination of sample sizes for animal experiments was based on our experience. We made efforts to minimize animal numbers in experiments, and basically 3 mice per group were used in an experiment.
Data exclusions	No data was excluded from analysis.
Replication	Most experiments were repeated at least twice to verify that experimental findings were reproducible.
Randomization	Randomization was not performed in this study.
Blinding	Blinding was not performed in this study.

Reporting for specific materials, systems and methods

We require information from authors about some types of materials, experimental systems and methods used in many studies. Here, indicate whether each material, system or method listed is relevant to your study. If you are not sure if a list item applies to your research, read the appropriate section before selecting a response.

Materials & experimental systems

n/a	Involved in the study	
☒	☐	Antibodies
☒	☐	Eukaryotic cell lines
☒	☐	Palaeontology and archaeology
☐	☒	Animals and other organisms
☒	☐	Clinical data
☒	☐	Dual use research of concern
☒	☐	Plants

Methods

n/a	Involved in the study	
☒	☐	ChIP-seq
☒	☐	Flow cytometry
☒	☐	MRI-based neuroimaging

Animals and other research organisms

Policy information about studies involving animals; ARRIVE guidelines recommended for reporting animal research, and Sex and Gender in Research

Laboratory animals	Balb/c and C57BL/6 mice were obtained from Japan SLC (Hamamatsu, Shizuoka, Japan). Dectin-2KO (1), CARD9KO (2) and LPLAT9 (LPCAT2) (loxP) (Acc.No.CDB0649K, https://large.riken.jp/distribution/mutant-list.html) (3) mice have been described previously. Mice were maintained under specific pathogen-free conditions and female mice were used at 8-12 weeks of age. Mice were housed in an ambient temperature- and humidity-controlled environment on a 12-hour light/dark cycle to mimic natural conditions.<br><br>(1) Saijo, S. et al. Dectin-2 Recognition of alpha-mannans and induction of Th17 Cell differentiation is essential for host defense against Candida albicans. Immunity 32, 681-691, doi:10.1016/j.immuni.2010.05.001 (2010).<br>(2) Hara, H. et al. The adaptor protein CARD9 is essential for the activation of myeloid cells through ITAM-associated and Toll-like receptors. Nat Immunol 8, 619-629, doi:10.1038/ni1466 (2007).<br>(3) Shindou, H. et al. Relief from neuropathic pain by blocking of the platelet-activating factor-pain loop. FASEB J 31, 2973-2980, doi:10.1096/fj.201601183R (2017).
Wild animals	No wild animals were used in the study.
Reporting on sex	We mainly used female mice in the study. In some cases, we compiled data using male and female mice as described in figure legends.
Field-collected samples	No field-collected samples were use.
Ethics oversight	All animal experiments were approved by the Animal Research Committee, Graduate School of Biostudies, Kyoto University (protocol number Lif-K20008).

Note that full information on the approval of the study protocol must also be provided in the manuscript.
